# Familial Hyperphosphatemic Tumoral Calcinosis

**DOI:** 10.1016/j.aace.2023.05.008

**Published:** 2023-06-01

**Authors:** Mohammad Saifuddin, Indrajit Prasad, Mirza Sharifuzzaman, Moinul Islam, Mobarak Hossain

**Affiliations:** Department of Endocrinology, Dhaka Medical College Hospital, Dhaka, Bangladesh

### Case Presentation

A 28-year-old man was referred because of nonspecific pain in the left lateral thigh. The patient stated that he experienced similar pain occasionally for the last 3 years for which he used took some medications, such as paracetamol or other pain killers, from the local pharmacy and pain sometimes relieved. There was no history of trauma at that site or associated fever or any other systemic symptoms. Examination revealed normal vital parameters, mild local pain on pressure with no other systemic abnormality. X-ray showed flecks of calcification in soft tissue of the left thigh (Fig.). Serum calcium, parathyroid hormone, and 25-hydroxyvitamin D levels were normal; however the serum phosphate level was extremely high (9.3 mg/dL; normal range, 1.7-4.5 mg/dL).

**Fig:**
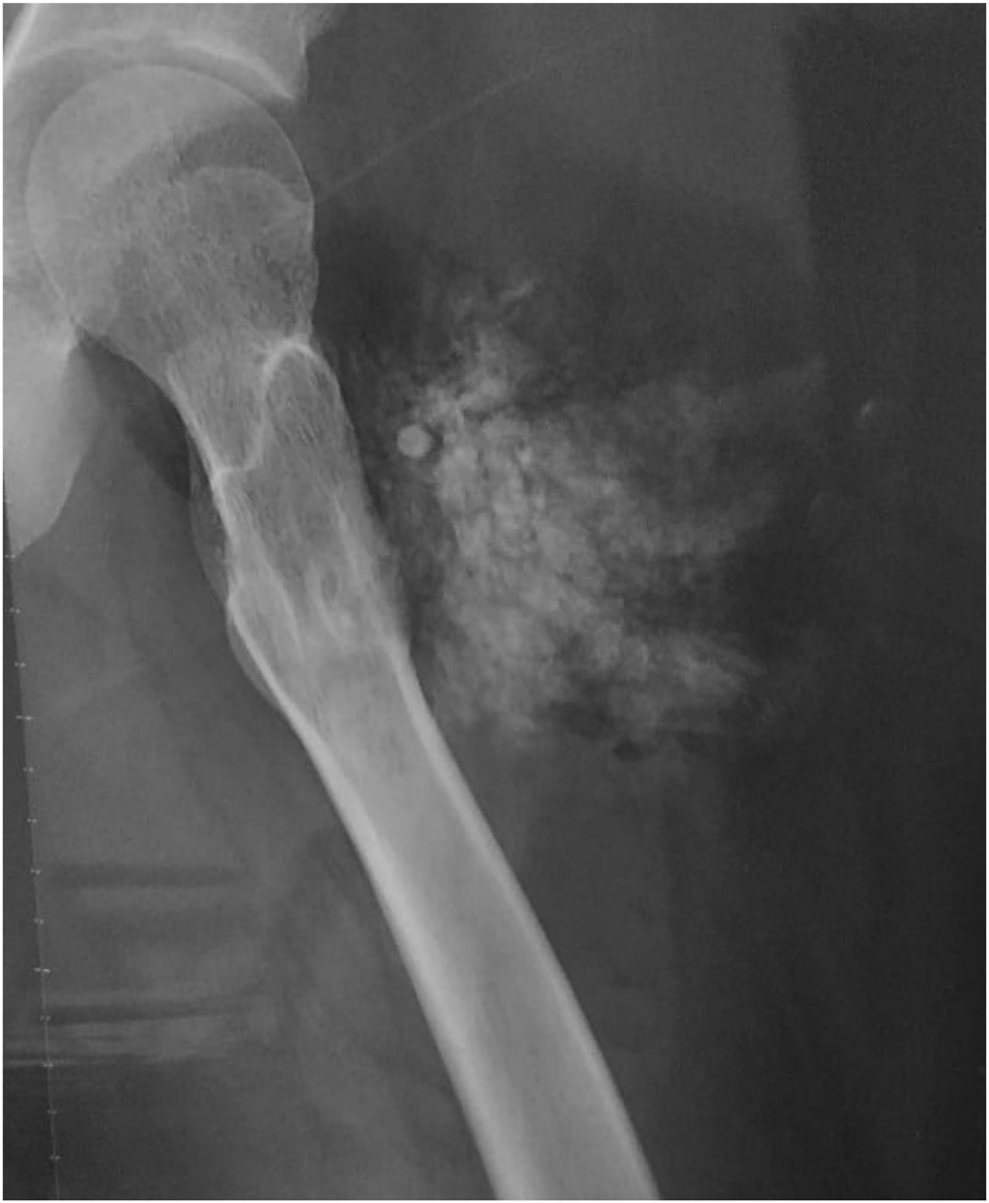

### What is the diagnosis?

#### Answer

Biopsy was performed to reach the final diagnosis of familial hyperphosphatemic tumoral calcinosis. Familial hyperphosphatemic tumoral calcinosis is an autosomal recessive disorder presented with hyperphosphatemia because of increase in proximal tubular phosphate reabsorption.^1^ The clinical manifestation is deposition of calcium phosphate adjacent to the joints. Clue to the diagnosis of familial hyperphosphatemic tumoral calcinosis is soft tissue calcification with hyperphosphatemia and normal calcium and parathyroid hormone levels. Fibroblast growth factor-23 and genetic markers are not available in Bangladesh. Pseudohypoparathyroidism, pseudopseudohypoparathyroidism, progressive osseous heteroplasia, fibrodysplasia ossificans progressiva, and porphyria cutanea tarda are the differential diagnoses. Management is integrative, including dietary restriction of phosphate (milk, cheese, eggs, and protein-rich food) and phosphate-lowering agents, such as aluminum hydroxide and sevelamer.^2^

## Disclosure

The authors have no multiplicity of interest to disclose.
